# A dating success story: genomes and fossils converge on placental mammal origins

**DOI:** 10.1186/2041-9139-3-18

**Published:** 2012-08-10

**Authors:** Anjali Goswami

**Affiliations:** 1 Department of Genetics, Evolution & Environment, and Department of Earth Sciences, University College London, Darwin Building, Gower Street, London, WC1E 6BT, UK

**Keywords:** Divergence estimates, Fossil record, Cretaceous-Paleogene mass extinction, Mammals

## Abstract

The timing of the placental mammal radiation has been a source of contention for decades. The fossil record of mammals extends over 200 million years, but no confirmed placental mammal fossils are known prior to 64 million years ago, which is approximately 1.5 million years after the Cretaceous-Paleogene (K-Pg) mass extinction that saw the end of non-avian dinosaurs. Thus, it came as a great surprise when the first published molecular clock studies suggested that placental mammals originated instead far back in the Cretaceous, in some cases doubling divergence estimates based on fossils. In the last few decades, more than a hundred new genera of Mesozoic mammals have been discovered, and molecular divergence studies have grown from simple clock-like models applied to a few genes to sophisticated analyses of entire genomes. Yet, molecular and fossil-based divergence estimates for placental mammal origins have remained remote, with knock-on effects for macro-scale reconstructions of mammal evolution. A few recent molecular studies have begun to converge with fossil-based estimates, and a new phylogenomic study in particular shows that the palaeontological record was mostly correct; most placental mammal orders diversified after the K-Pg mass extinction. While a small gap still remains for Late Cretaceous supraordinal divergences, this study has significantly improved the congruence between molecular and palaeontological data and heralds a broader integration of these fields of evolutionary science.

## Background

As George Gaylord Simpson [1] detailed in his ground-breaking volume, one of the main contributions of the fossil record to the modern synthesis is primary data on the tempo of evolution. In recent decades, this aspect of fossil data has been leveraged for calibrating molecular estimates of clade divergence times. Concurrently, there has been expanding use of molecular data in palaeobiological studies (for example, [2]), and linking of fossils and embryos in studies of evolutionary development (for example, [3]). Yet, these examples of integration between fields of evolutionary science are still rare, and fossils are often excluded from macroevolutionary analyses beyond palaeobiology because of the complexities associated with including extinct taxa of uncertain phylogenetic affinity or with incorporating incomplete data. It has also been suggested that fossil data will likely have little impact on reconstructions of evolution in extant lineages (for example, [4,5]), despite extensive evidence to the contrary (for example, [6,7]). One of the most persistent question marks concerning the quality of fossil data has come from molecular dating studies themselves, which can differ from fossil-based divergence time estimates by tens to hundreds of millions of years and are regarded by many as showing the poor quality of the fossil record [8].

For this reason, many palaeobiologists were pleased when a recent study [9] using a vast genomic dataset to reconstruct divergence dates for major placental mammal clades, found extensive congruence with the fossil record. Indeed it appears that most placental mammal orders originated and diversified after the demise of the non-avian dinosaurs during the Cretaceous-Paleogene (K-Pg) mass extinction 65.5 million years ago (mya), as palaeomammalogists have long maintained. The timing of this radiation has been a contentious issue since the first published molecular clock studies of placentals pushed the divergence times for many clades deep into the Cretaceous [10-12], in some cases nearly doubling the time between molecular origins and first appearance in the fossil record.

## Accurate dating is crucial for understanding the last mass extinction

**Figure 1:**
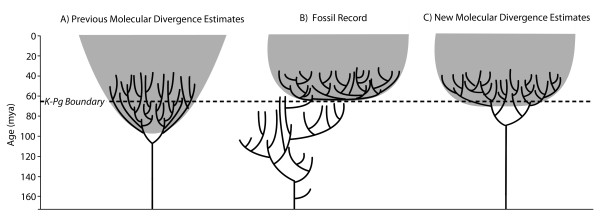
** Schematic comparison of placental mammal divergence estimates based on: (A) previous molecular divergence studies **[4,10]**; (B) the fossil record of eutherians, including placentals**[14,15]**; and (C) the recently published divergence estimates from the phylogenomic analysis of dos Reis *et al. ***[9]. The shaded grey areas denote the period during which significant intraordinal divergences within placental mammals occur, which, for B and C, correspond with large increases in placental mammal taxonomic diversity recorded in the fossil record. Note that these representations are for general pattern only, and do not include estimates of error, which were particularly large in the early molecular clock analyses [20]. Many molecular divergence studies show a similar pattern as A, with several placental inter- and intraordinal divergences in the Cretaceous [4,11,13], while some others approach fossil-based estimates [9,17-19].

Resolving the timing of the placental mammal radiation is crucial for understanding the magnitude and selectivity of the K-Pg mass extinction, as well as factors that shaped the evolution of mammals and, more generally, the modern biota. Most molecular divergence studies until now have favored a ‘Short Fuse Model’, in which the major clades originated and diversified long before the K-Pg boundary, with some even suggesting that the extinction of non-avian dinosaurs had little to no effect on the evolution of extant clades [4]. Others have supported a ‘Long Fuse Model’, in which major clades originated long before their first appearance in the fossil record, but did not diversify extensively until after the K-Pg extinction cleared valuable niche space for mammals to occupy [13]. The palaeontological evidence has been consistently in support of an ‘Explosive Model’, wherein the major clades originated and diversified near the K-Pg boundary [14,15]. However, as stem members of several placental orders can be identified within a few million years of the K-Pg boundary, most palaeontologists accept that some placental lineages may well extend into the Late Cretaceous, as suggested by the Long Fuse Model [16]. Over the last few years, molecular divergence estimates have been steadily moving closer to those supported by palaeontological data [17,18]. Another recent analysis of a molecular supermatrix also reconstructed most intraordinal divergences near the Cretaceous-Paleogene (K-Pg) boundary [19], but the dos Reis *et al.*[9] study goes further still in closing the long-standing gap between molecular data and fossils (Figure 1).

## Mind the gap

Many hypotheses have been suggested for the persistent discrepancy between molecular and fossil-based estimates, and a primary one is incomplete sampling in the fossil record. Rock availability and collection effort can drive apparent patterns of taxonomic diversity [21,22], but statistical models suggest that preservation alone is unlikely to explain the difference in divergence estimates for placental mammals [23]. The relatively poor sampling of Mesozoic mammal fossils from the large southern continents may be a confounding factor, but current data offer little support for a southern ‘Garden of Eden’ for placental mammals [14,15]; but see [25]. On the molecular side, the effects of body size, population fluctuations, overlapping generations and other life history and ecological factors have been shown to affect estimates of substitution rates, potentially misleading divergence estimates [26,27].

The new study by dos Reis *et al.*[9] suggests that previous molecular clock studies were misled by inadequate molecular data, poor quality control and treatment of fossil calibrations, and overly simplistic treatment of the variation in molecular data. The immense amount of data (36 nuclear genomes and 274 mitochondrial genomes) used in this study was certainly the most obvious improvement, representing an increase of multiple orders of magnitude (in terms of genes sampled) over previous studies. Perhaps even more important was the use of more accurate fossil data, following the increasing participation of palaeobiologists in establishing calibration dates (for example, [9,28,29]), as well as a more realistic use of those dates as ‘soft’ bounds or probability distributions, and not as invariant minima [30]. Indeed, much of the offset in previous studies may have been due to inappropriate treatment of fossil data, such as using single calibrations from poorly sampled intervals [31] or incorrectly assuming that Cretaceous eutherians represent extant clades [10]. In addition, methodological innovations in the approach used by dos Reis *et al.*[9] allowed for better treatment of branch length and rate variance, which will be even more important as next-generation sequencing continues to expand the use of genomic data in phylogenetic and other evolutionary analyses.

## Fossils are more than calibration points

The utility of fossils extends beyond their use as first appearance events. Some studies have attempted to model clade origins based on present and past diversity, as well as sampling intensity, with one such analysis concluding that Primates likely originated in the Cretaceous [32]. Quantifying rates of phenotypic character change across fossil-dominated phylogenetic trees for placentals has also suggested that there is no significant change immediately before and after the K-Pg boundary [33]. However, analyses of this nature are preliminary and limited by the accuracy of available phylogenies, which, in the case of mammals, usually focus either on Cretaceous or more recent Cenozoic taxa. Perhaps the most problematic aspect of these approaches at present is that placental mammals from the Paleocene (the ten-million year interval immediately following the K-Pg extinction) are phylogenetically poorly resolved and thus excluded in most studies. Ongoing work should eventually resolve early Cenozoic mammal relationships, allowing for integration of a broader range of fossil data into studies of mammalian evolutionary rates and clade origins.

New methods will require good fossil data, and in the intervening 14 years since the publication of the first extensive molecular clock estimates of mammal divergence times [10], more than 100 new genera of Mesozoic mammals have been discovered [34]. Although none of these taxa are currently well-supported as stem representatives of extant placental lineages [14,15]; but see [25,35], they provide important data on the effect of the K-Pg extinction on mammals and cast doubt on extant-only based reconstructions, which suggest that at least 43 placental lineages survived that event [4]. Fossil evidence demonstrates that there was a diverse mammalian fauna leading up to the K-Pg extinction, which included eutherians and metatherians (the clades that include placentals and marsupials, respectively), as well as the taxonomically-depauperate, but still extant egg-laying monotremes. In addition, the latest Cretaceous saw cosmopolitan dryolestoids and multituberculates, as well as gondwanatheres and other clades with southern or more restricted distributions. Most of these clades survive the K-Pg extinction, but in limited numbers. Other than the extant clades, only multituberculates recovered or maintained, according to [36], high levels of diversity in the Paleogene. Between Eutheria and Metatheria, only five lineages (Herpetotheriidae, Cimolestidae, Adapisoriculidae, Leptictidae [+*Gypsonictops*, and *Protungulatum*) are known to have survived the K-Pg extinction, while many others perished [14,15,37]. The dos Reis *et al.* study [9] adds another dozen or so lineages to the list of survivors, but it is clear that evidence from fossils and molecules are converging on a model where a small number of mammalian lineages survive the K-Pg extinction and diversify soon afterwards.

## The fuse remains cryptic

Although the gap has closed substantially, disagreement between the most recent molecular divergence time estimates and the fossil record persists. Intraordinal divergence estimates now nearly replicate the fossil record of placental mammals, but the oldest interordinal divergences continue to predate the first fossil occurrences by approximately 20 million years. The fuse, relative to the fossil record, is almost 50 million years shorter than in the first molecular clock studies, and may change further. At the moment, however, this suggests that nearly 25% of placental mammal history remains unrepresented in the fossil record, if none of the known Cretaceous eutherians fall along the crown’s Mesozoic fuse. This remaining gap emphasizes a continuing need for palaeontological exploration, as was highlighted by the recent discovery of an approximately 160 million-year-old eutherian [38]. The fossil record and molecular divergence studies have both improved dramatically in recent years and continue to demonstrate that there is much more to learn about mammal evolution.

## Conclusions

The great strides that have been made in resolving the timing of placental mammal origin may also bode well for the many other contentious gaps between molecular divergence estimates and the fossil record. The timing of origin for eukaryotes is a notable area of ongoing debate, with some molecular estimates and fossil-based estimates differing by more than a billion years, (but see [39]). The origin of animals (Metazoa) is no less controversial, with some molecular dates predating the fossil evidence for a ‘Cambrian Explosion’ by 800 million years, although most studies find the gap to be a mere 100 million years [40,41]. Studies such as the one by dos Reis *et al.*[9] demonstrate that better integration of molecular and palaeontological data is essential for a more accurate understanding of the patterns and processes underlying organismal evolution. Hopefully, this progress in divergence time estimates will encourage further synthesis of the diverse sources of data available to evolutionary biologists. It is long overdue.
